# Using Higher Diffraction Orders to Improve the Accuracy and Robustness of Overlay Measurements

**DOI:** 10.3390/mi16030347

**Published:** 2025-03-19

**Authors:** Shaoyu Liu, Yan Tang, Xiaolong Cheng, Yuliang Long, Jinfeng Jiang, Yu He, Lixin Zhao

**Affiliations:** 1 National Key Laboratory of Optical Field Manipulation Science and Technology, Chinese Academy of Sciences, Chengdu 610209, China; liushaoyu23@mails.ucas.ac.cn (S.L.); tangyan@ioe.ac.cn (Y.T.);; 2 State Key Laboratory of Optical Technologies for Micro-Fabrication, Institute of Optics and Electronics, Chinese Academy of Sciences, Chengdu 610209, China; 3 Institute of Optics and Electronics, Chinese Academy of Sciences, Chengdu 610209, China; 4 University of Chinese Academy of Sciences, Beijing 100049, China

**Keywords:** diffraction-based overlay, lithography, optical metrology, gratings

## Abstract

This paper introduces a method for improving the measurement performance of single wavelength overlay errors by incorporating higher diffraction orders. In this method, to enhance the accuracy and robustness of overlay error detection between layers, the measurement errors introduced by empirical formulas are corrected by incorporating higher diffraction orders, based on the differences in the light intensity difference curves for different diffraction orders. This method also expands the range of available wavelengths for selection. The introduction of specially designed overlay error measurement markers enhances the diffraction efficiency of higher diffraction orders to overcome the issue of their weak light intensity, making them difficult to utilize effectively. This paper first conducts a theoretical analysis using scalar diffraction theory, and then demonstrates the feasibility of the design through vector diffraction simulations and optical path simulations. The resulting two-layer marker structure is simple and compatible with existing measurement systems, showing tremendous potential for application at advanced process nodes.

## 1. Introduction

The fabrication of nanoscale devices typically involves multiple lithography steps. In each lithography step, the pattern on the mask must be precisely transferred onto the wafer at the correct position, ensuring that the positional error relative to the previous lithography step remains within an acceptable range. This process is known as overlay. As one of the three key performance metrics of a lithography machine, overlay error characterizes the positional deviation between different lithographic layers. Generally, the overlay error should be kept below 20–30% of the critical dimension (CD) [1]. According to the International Roadmap for Devices and Systems (IRDS), the measurement error of overlay must be controlled at the sub-nanometer level [2] now.

**Figure 1:**
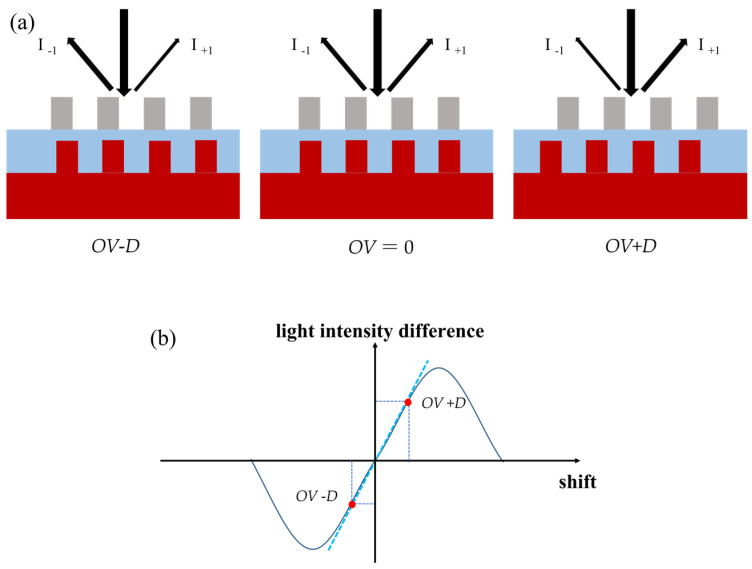
(**a**) The relation between overlay error (*OV*) and shift. The intensity difference of ±first-order diffracted light becomes nonzero due to the presence of overlay. (**b**) The light intensity difference curve varying with shift.

According to different technological approaches, overlay detection methods are categorized into image-based overlay (IBO) and diffraction-based overlay (DBO). IBO utilizes a high-resolution imaging system to capture images of the alignment marks, followed by image processing algorithms to extract overlay error. However, this method is limited by imaging resolution, making it challenging to apply in advanced process nodes. DBO [3], on the other hand, extracts information by analyzing the optical diffraction signals from the alignment marks, typically based on ±first order diffracted light. The principle is illustrated in the Figure 1. When no overlay error exists, the intensity difference between the ±first order diffraction signals is zero. When a positional shift occurs, the intensity difference deviates from zero. The relationship between intensity difference and shift follows a sinusoidal-like curve, with a well-defined linear region within a certain range. By introducing a predefined offset *D* and applying an empirical formula Equation (3), overlay error can be rapidly extracted.

(1) $$I_{-D}=k(OV-D)$$

(2) $$I_{+D}=k(OV+D)$$

(3) $$OV=D\cdot \frac{I_{+D}+I_{-D}}{I_{+D}-I_{-D}}$$

Since the intensity difference curve is influenced by multiple factors, such as wavelength, diffraction order, and mark structure, significant measurement errors may occur. Therefore, improving accuracy and robustness is the primary challenge faced by DBO. Researchers have explored various approaches, including wavelength selection [4,5], multi-wavelength utilization [6,7], optimization of mark structure design [8,9,10,11], and error signal analysis and compensation [12,13,14]. Among these factors, mark structure plays a crucial role in determining measurement performance, as it affects the measurement capabilities at different wavelengths, the energy distribution and propagation direction of each diffraction order, and the magnitude of error signals. Thus, mark structure is one of the key factors influencing measurement accuracy.

Therefore, to improve measurement accuracy and robustness, this paper proposes introducing the third diffraction order and optimizing the mark design using a diffraction efficiency enhancement method to increase the diffraction efficiency of this order. Compared with traditional methods, (1) the diffraction efficiency of the third diffraction order is significantly improved while ensuring that the first-order efficiency remains at a similar level; (2) the proposed method maintains good compatibility with existing detection systems without altering the current measurement principle; and (3) it corrects the issue of large errors at certain wavelengths, which are otherwise difficult to use, thereby expanding the usable spectral range. The structure of this paper is as follows: 1. Introduction, 2. Principle, 3. Simulation, 4. Summary.

## 2. Principle

### 2.1. Resistance of Higher Diffraction Orders to Asymmetric Deformation

**Figure 2:**
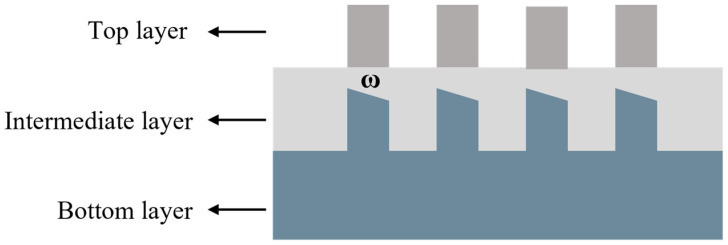
The linear asymmetric deformation of the bottom mark induced by chemical mechanical polishing.

The bottom grating is prone to linear asymmetric deformation after chemical mechanical polishing (CMP), as shown in the Figure 2 below. Such asymmetric deformation introduces additional error signals, which in turn affect the measurement results. Yang [12] derived the error signal using scalar diffraction theory and obtained the following result:

(4) $${\left|C_{+m}\right|}^{2}-{\left|C_{-m}\right|}^{2}=\frac{4}{{\left(w-2\pi m\right)}^{2}}\sin^{2}(\frac{w-2\pi m}{4})-\frac{4}{{\left(w+2\pi m\right)}^{2}}\sin^{2}(\frac{w+2\pi m}{4})$$

Simplifying the formula yields the following [12]:

(5) $${\left|C_{+m}\right|}^{2}-{\left|C_{-m}\right|}^{2}=\left\{ \begin{array}{l} \frac{32\pi }{\frac{w^{3}}{m}-8\pi^{2}wm+\frac{16\pi^{4}}{w}m^{3}}\cos^{2}(\frac{w}{4})m=1,3,5\ldots \\ \frac{32\pi }{\frac{w^{3}}{m}-8\pi^{2}wm+\frac{16\pi^{4}}{w}m^{3}}\sin^{2}(\frac{w}{4})m=2,4,6\ldots \end{array}\right.,$$

In this context, *m* represents the diffraction order, C_+*m*_ and C_−*m*_ represent the Fourier coefficient of the mark grating amplitude, the square difference between C*_+m_* and C_−*m*_ is defined as the error signal, and *w* is the coefficient for the linear deformation of the mark relate to process. As the diffraction order increases, the error signal rapidly decreases. Therefore, incorporating higher diffraction orders enhances the resistance to asymmetric deformation.

### 2.2. Principle of Multiple Diffraction Order Sharing

**Figure 3:**
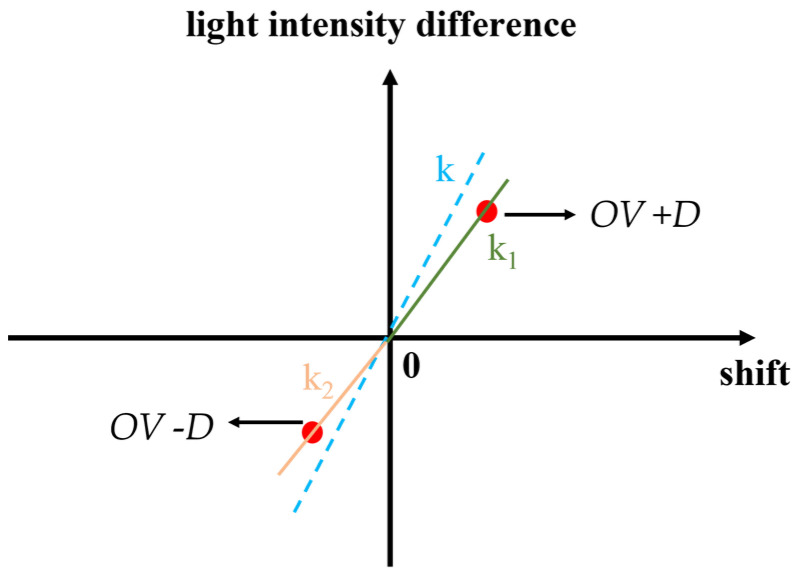
Two points on the intensity difference curve form two lines with different slopes relative to the origin. Both lines differ from the straight line assumed by the empirical formula:

The commonly used empirical formula for overlay metrology relies on a local linearity assumption [15], but in practice an error value often exists, as shown in the Figure 3 below. The two points on the curve do not always perfectly align with the linear assumption relative to the origin. More often, these two points form two straight lines with different slopes with respect to the origin. Therefore, the empirical formula can be rewritten in the following form.

(6) $$I_{-D}=k_{1}(OV-D),$$

(7) $$I_{+D}=k_{2}(OV+D),$$

(8) $$OV_{M}=D\cdot \frac{(k_{1}+k_{2})OV}{(k_{2}-k_{1})OV+(k_{1}+k_{2})D}-D\cdot \frac{(k_{1}-k_{2})D}{(k_{2}-k_{1})OV+(k_{1}+k_{2})D},$$

In this context, *OV_M_* represents the measured overlay error value. The second term represents the maximum source of error. In the second term, *k*_1_ and *k*_2_ are related to the intensity difference curve. Therefore, Equation (8) can be simplified into the form of Equation (9).

(9) $$OV_{M}=OV-C(\lambda ,m),$$

In Equation (9), *OV* is the actual overlay error value, and C is the error term related to the wavelength (*λ*) and diffraction order (*m*). In previous work [6,7], multiple wavelengths were often used for self-referencing to reduce measurement errors and enhance robustness. In this paper, multiple diffraction orders are used instead, as the intensity differences between different diffraction orders are similar to those of different wavelengths, and since higher-order diffracted light has better resistance to asymmetric deformation at the top, higher-order diffraction light can be used to correct measurement errors, leading to more accurate and robust measurement results.

### 2.3. Diffraction Efficiency Enhancement Theory

Although incorporating higher-order diffracted light yields the benefits mentioned above, the energy of higher-order diffracted light is weak, making it difficult to use directly. In overlay detection, the markers are designed as a structure of two stacked gratings, where both have the same line width and period. Therefore, when designing the markers, it is only necessary to consider a single-layer grating. The subdivision structure method is a commonly used approach for enhancing diffraction efficiency. In this method, sub-grating structures are added to the original grating, allowing for the enhancement of the diffraction efficiency of one or several specific diffraction orders.

The following equation represents the diffraction field expression derived based on scalar diffraction theory [16]:

(10) $$U_{m}(h,f_{i},x_{i},N)=\left\{ \begin{array}{ll} 1+\sum_{i=1}^{N}[f_{i}\exp(-j\frac{4\pi h}{\lambda })-f_{i}], & m=0\\ \sum_{i=1}^{N}\frac{\sin(m\pi f_{i})}{m\pi }[\exp(-j\frac{4\pi h}{\lambda })-1]\exp(-j\frac{2m\pi }{p}x_{i}), & m=\pm 1,\pm 2\ldots \end{array}\right.$$

Further, the diffraction efficiency *η_m_* expressions for each diffraction order are as follows; it represents the ratio of the intensity of each diffraction order to the incident light intensity [16]:

(11) $$\eta_{m}(h,f_{i},x_{i},N)=\left\{ \begin{array}{ll} 1-4\sin^{2}(\frac{2\pi }{\lambda }h)(1-\sum_{i=1}^{N}f_{i})\sum_{i=1}^{N}f_{i}, & m=0\\ \frac{4}{{\left(m\pi \right)}^{2}}\sin^{2}(\frac{2\pi }{\lambda }h){\left|\sum_{i=1}^{N}\sin(m\pi f_{i})\exp(-j\frac{2m\pi }{p}x_{i})\right|}^{2}, & m=\pm 1,\pm 2\ldots \end{array}\right.$$

In this case, *h* represents the grating ridge height, *f_i_* is the duty cycle of the *i-th* structure, and *x_i_* is the center position of the *i-th* structure. Therefore, by adding sub-grating structures with specific line widths at certain positions, an enhancement effect can be achieved. Expanding the efficiency expression of the higher diffraction orders for *N* = 2 yields the following form:

(12) $$\eta_{m\neq 0}=\frac{4}{{\left(m\pi \right)}^{2}}\sin^{2}(\frac{2\pi }{\lambda }h)\left\{\sin^{2}(m\pi f_{1})\cos^{2}(\frac{4m\pi }{p}x_{1})+2\sin(m\pi f_{1})\sin(m\pi f_{2})\cos\left[\frac{2m\pi }{p}(x_{1}+x_{2})\right]+\sin^{2}(m\pi f_{2})\cos^{2}(\frac{4m\pi }{p}x_{2})\right\}$$

**Figure 4:**
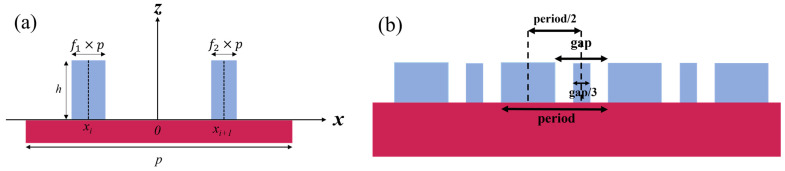
(**a**) Diagram of the diffraction efficiency enhancement structure. (**b**) Third order diffraction efficiency enhancement structure obtained through design.

To ensure the ±m order diffraction efficiencies are identical, the center positions of the two grating ridges must be opposite (as shown in the coordinate system in Figure 4a). Furthermore, since measurement often involves rotating the mark by 180° for re-measurement to reduce TIS (tool induced shift), it is necessary to satisfy 180° rotational symmetry [1].

Since the first diffraction order inherently has higher light intensity, this forms the basis for traditional methods using the ±1 orders for measurements. In this work, the third diffraction order is chosen for enhancement. The structure with *N* = 2 is selected, meaning only one sub-grating structure is added. The enhancement condition can be simplified as follows: dividing the fine sub-grating structure into *m* equal parts enhances the diffracted signal for the *m-th* diffraction order [17]. The schematic of the designed structure is shown in Figure 4b. To enhance the third order diffraction, the linewidth of the sub-grating structure is set to one third of the gap width, with its center positioned at the center of the gap.

## 3. Simulation

Since the above theory is derived based on scalar diffraction theory without considering the effects of light polarization, and the marking structure is relatively small, the error is significant, making it suitable only as a design reference. The following will further demonstrate the effectiveness of this method through the rigorous coupled-wave analysis (RCWA) [18,19,20,21,22] and ray-tracing simulations.

**Figure 5:**
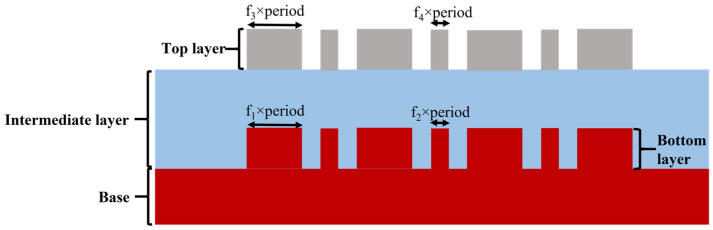
The designed DBO mark with enhanced third order diffraction efficiency.

By optimizing the reference mark and selecting the incident wavelength [4,5], considering the actual processing conditions and the aforementioned design theory, the initial structural parameters were obtained as shown in the Table 1, the schematic diagram of the structure is shown in Figure 5. In the simulation, the mark period was 2000 nm, and the incident wavelength was 520 nm. An off-axis illumination method was used, with an incident angle of 36°, and the polarization direction of the incident light was parallel to the grating lines. The preset offset *D* was 20 nm.

### 3.1. RCWA Simulation

**Figure 6:**
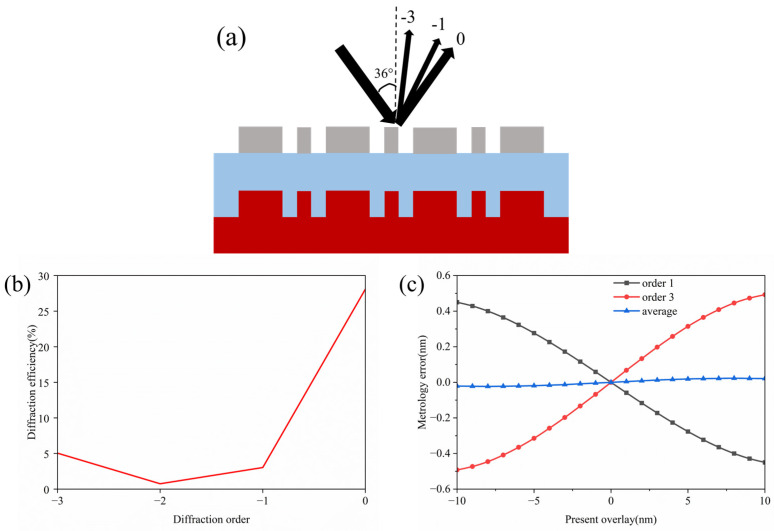
(**a**) The settings of the illumination methods in the simulation; (**b**) the diffraction efficiency of the simulated structure; (**c**) the measurement error utilizing each diffraction order in the simulation.

Assuming an ideal case where the mark has no deformation and the light source is incident in the direction shown in Figure 6a, the diffraction efficiency of each order is depicted in Figure 6b. The simulation results indicate that, with the proposed design, the 3rd order diffraction efficiency was enhanced by more than 6 times, demonstrating the effectiveness of this method. Figure 6c shows the measurement error curve under a preset overlay error of 10 nm. Here, the measured signal was substituted into the empirical formula to obtain the measured value (*OV_M_*), which was then subtracted by the preset value (*OV*) to determine the metrology *error*:

(13) $$error=OV_{M}-OV,$$

When using a single diffraction order, the maximum metrology error reached nearly 0.5 nm, which is unacceptable in practical applications. However, when the first and third diffraction orders were used together, the measurement error was well corrected, with a maximum measurement error of only 0.02 nm, not exceeding 0.2% of the preset error value.

To better reflect real conditions, the measurement errors caused by the mark were abstracted into two types: top asymmetrical deformation and sidewall asymmetrical (SWA) deformation. For the two-layer grating of the mark, the bottom layer underwent multiple processing steps, making it more susceptible to deformation. As shown in the figure, the two types of deformation for the bottom grating are illustrated. In the simulation, the maximum top tilt angle *α* was set to 1°, corresponding to height values of $h_{1}^{\prime }=19.2\;\mathrm{nm}$. and $h_{2}^{\prime }=5.2\;\mathrm{nm}$. The maximum sidewall angle *θ* was set to 7°, with the corresponding deformation distances of $b_{1}^{\prime }=b_{2}^{\prime }=12.3\;\mathrm{nm}$.

**Figure 7:**
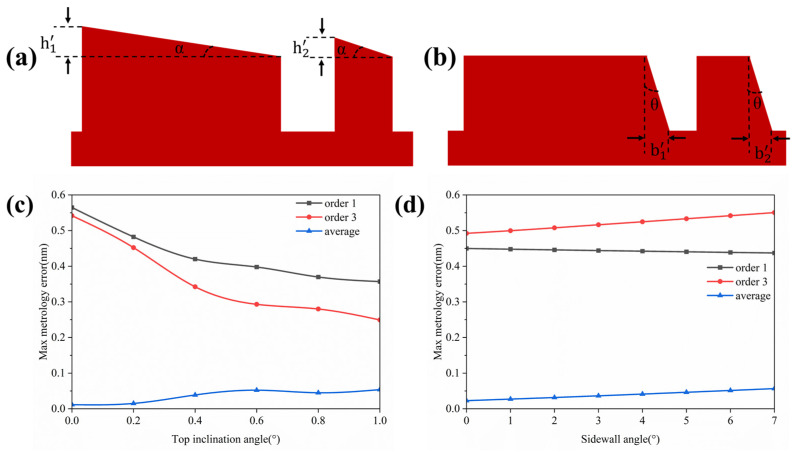
(**a**) The linear asymmetric deformation generated labeled at the top of the bottom section; (**b**) the sidewall tilt angle generated labeled in the bottom section; (**c**) the maximum metrology error under different top tilt angles; (**d**) the maximum metrology error under different sidewall angles.

Figure 7c,d shows the maximum metrology error curves obtained as the process errors increase. Due to the presence of process errors, (1) the centroid of the mark structure shifted, generating an additional error signal [13]. (2) As discussed in Section 2.2, solving the overlay error value using an empirical formula inevitably introduces some error, which originates from the linear assumption. The additional error signal caused by process errors may counteract this error signal, thereby correcting the error and reducing the metrology error. This situation is illustrated in Figure 7c. From the curves, it can be seen that the use of the 3rd diffraction order exhibited better resistance to the top angle, which is consistent with the theory. By combining both types of asymmetric errors, the 1st and 3rd diffraction orders shared the measurement, significantly reducing the metrology error. The maximum metrology error did not exceed 0.057 nm, making this wavelength suitable for calibration.

### 3.2. Optical Detection System Simulation

**Figure 8:**
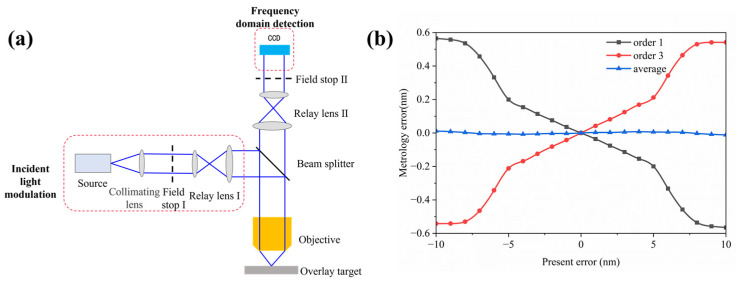

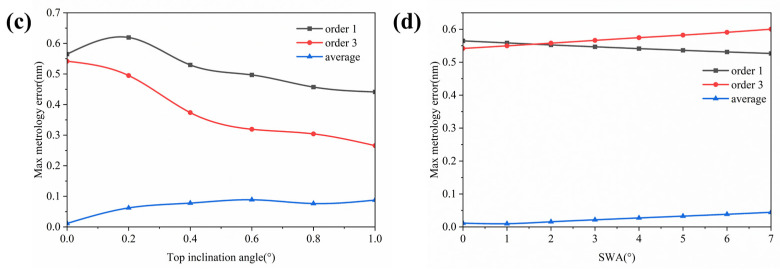
(**a**) The schematic diagram of the frequency-domain measurement system; (**b**) the metrology error curve obtained in the absence of asymmetric deformations; (**c**) the maximum metrology error under different top tilt angle conditions obtained through the optical path simulation; (**d**) the maximum metrology error under different sidewall angle conditions obtained through the optical path simulation.

To obtain more accurate measurement results, a frequency-domain detection system wasused for the optical path simulation; the structure of the measurement system is shown in Figure 8a. Off-axis illumination was employed, with the light incident at a 36° angle onto the DBO marking. The polarization direction was parallel to the grating lines. After passing through a beam splitter and relay lens, the diffracted light was collected by the charge-coupled device (CCD), and the intensity of each diffraction order was measured at the pupil plane. Subsequent calculations were performed using an empirical formula.

The measurement results of the ideal case without process errors, with a maximum metrology error of 0.012 nm, are shown in Figure 8b,. The two types of asymmetric deformations from the previous section were placed at the DBO marking location for remeasurement. Figure 8c shows the maximum metrology error related to the top linear tilt, and Figure 8d shows the maximum metrology error related to the sidewall tilt, with both having a maximum metrology error not exceeding 0.09 nm. Compared with the RCWA simulation results from the previous section, consistent measurement results are obtained, further validating the effectiveness of this method.

## 4. Summary

Based on the characteristic that high diffraction orders have excellent resistance to partial asymmetric deformations of the marks, this paper proposes a DBO mark design method based on subdivided structures, which makes the utilization of high diffraction orders possible. The reasons for the excellent performance of high diffraction orders and the design principles of the DBO mark are explained using scalar diffraction theory. The principle of improving measurement performance by utilizing multiple diffraction orders is explained through an improved empirical formula. Finally, both the RCWA algorithm and optical path simulation confirm the effectiveness of this method, providing a new perspective for detecting lithographic overlay.

## Figures and Tables

**Table 1:** Simulation set.

Layer	Material	Height/nm	Critical Dimension/nm	
Bottom layer	Si	100	f_1_ × period (f_1_ = 0.55)	
			f_2_ × period (f_2_ = 0.15)	
Intermediate layer	SiO_2_	230		
	
Top layer	Photoresist [23]	100	f_3_ × period (f_3_ = 0.55)	
			f_4_ × period (f_4_ = 0.15)	

## Data Availability

The original contributions presented in the study are included in the article, further inquiries can be directed to the corresponding author.
